# Evaluation of glutathion peroxidase activity, trace minerals and weight gain following administration of selenium compounds in lambs

**Published:** 2017-06-15

**Authors:** Pedram Yaghmaie, Aligholi Ramin, Siamak Asri-Rezaei, Asghar Zamani

**Affiliations:** 1 *DVSc Candidate, Department of Internal Medicine and Clinical Pathology, Faculty of Veterinary Medicine, Urmia University, Urmia, Iran; *; 2 *Department of Internal Medicine and Clinical Pathology, Faculty of Veterinary Medicine, Urmia University, Urmia, Iran;*; 3 *Department of Nano Chemistry, Faculty of Science, Urmia University, Urmia, Iran.*

**Keywords:** GPX, Lamb, Nanoselenium, Sodium selenite, Weight

## Abstract

Blood selenium and trace minerals play an important role in animal’s health and production. The aims of this study were to determine selenium effect on blood glutathione peroxidase (GPX) activity, trace minerals and weight gain in lambs. Twelve female Makuei breed were studied for 63 days in groups of control, nanoselenium (NanoSe) and sodium selenite (NaSe). Mean concentrations of GPX, Cu and Fe in selenium supplemented groups were higher than in control group but the differences were not significant. Mean GPX and selenium was significant among the bleeding times, for Cu and Zn significant occasionally while not for weight gain. The percentages of weight gain in groups were 34.20, 38.90 and 36.30, respectively, which was not different. The individual comparison of parameters among groups showed differences for GPX, selenium and Cu. Correlations were observed between weight & Fe, weight & GPX, weight & selenium, Zn & Fe and GPX & selenium in NanoSe group. Weight gain showed negative correlations with Fe and positive correlation with GPX. In conclusion, selenium compounds increased GPX activity and selenium in which it was predominant in NanoSe than in NaSe group. Selenium compounds showed no effects on Cu, Zn and Fe but caused weight gain to increase. NanoSe revealed correlations between weight gain, GPX, Fe and selenium and was preferable to NaSe. Thus, the effect of NanoSe on reducing the oxidative stress and increased weight gain was acceptable and probably an option to NaSe administration in lambs.

## Introduction

Blood selenium and macro-minerals play an important role in animals’ health, production and reproduction.^1^ Selenium compounds activate glutathione peroxidase enzyme, which is responsible for reducing and eliminating oxidative stress^2^ resulting in an increase in immunity and prevention of diseases and reproductive disorders.^3^ Selenium compounds may interference with the meta-bolism of some trace minerals such as Zn, Fe and Cu that causes a decrease in their absorption.^4^^-^^6^ Despite progress in resolving selenium deficiency in lambs, efforts are made to introduce new compounds with high efficiency and less side effects to enhance animals’ production.^7^

Selenium nano-particles (NanoSe) have efficiency on growth, production, reproduction performances and immunity system.^8^ Their administration showed an increase in ruminal fermentation and digestion^9^ and improvements in feed intake,^10^ and therefore, their effect is more appropriate than sodium selenite (NaSe).^11^ Scientists agree that the role of NanoSe in glutathione peroxidase (GPX) activity^12^ improves blood components,^13^ urea, protein and blood creatinine^14^ and is more suitable than organic and inorganic selenium. Meanwhile, the toxic effect of NanoSe is less than 3 g kg^-1 ^of food dry matter which is lower than NaSe,^15^ indicating that it would be a suitable replacement for NaSe.

Despite the improvements in the use of NanoSe in animals, further studies are required to reveal the amounts of glutathione peroxidase activity, interference with trace minerals absorption and weight gain in lambs. The objectives of the current study include: comparison of glutathione peroxidase activity and selenium level following administration of NanoSe and NaSe, comparison of serum Cu, Fe and Zn concentrations in lambs receiving selenium compounds and evaluation of weight gain in lambs with selenium compounds.

## Materials and Methods

Twelve female Makuei lambs were divided into three groups (n = 4) as control, NanoSe and NaSe groups. The mean age and standard error for groups was 63.50 ± 1.30, 59.00 ± 1.20 and 69.80 ± 2.60 days, respectively, and for weight was 20.40 ± 1.03, 19.60 ± 0.95 and 22.25 ± 0.75 kg, respectively. The mean overall age and weight was 64 days and 20.70 kg, respectively. Lambs were mixed in flock with ewes and fed hay and lucerne on pasture. They were clinically examined and appeared normal.

Lambs were initially bled and weighed before the experiment. Recommended dose for NanoSe (Merck, Germany) with no side effects is 0.10 mg kg^-1 ^of live weight.^9^ The amount of NanoSe and NaSe (Merck, Darmstadt, Germany) was calculated based on the weight of each lamb and was divided into seven parts, administered daily up to seven days. The control lambs received water only. Blood samples were taken before administration of selenium compounds and then weekly until 63 days (10 times) of trial. Five mL blood was prepared into two tubes of with and without EDTA for hematological and biochemical tests, respectively. Weight gain was also measured weekly up to nine weeks.

Blood selenium concentration was measured by atomic absorption (AA6800, Shimatzu, Tokyo, Japan) using selenium lamp. Blood GPX activity was evaluated by commercial kit (Biorex, Antrium, UK) in the spectrophotometer and Cu, Zn and Fe by auto-analyzer machine (BT-1500; Biotecnica, Rome, Italy) and relevant kits (Pars Azmon, Tehran, Iran).

SPSS statistical program (version 17; SPSS Inc., Chicago, USA) was used for all analyses. ANOVA was used to determine the significance of weekly variation in mean concentrations of all groups and parameters. Pearson correlation was run to determine the relationships among parameters, mainly on blood selenium and weight gain. 

## Results

**Fig. 1:**
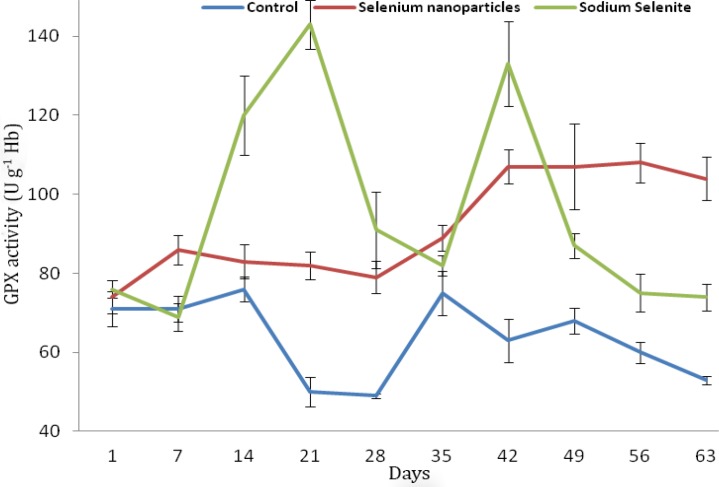
Mean comparison of blood glutathione peroxidase activity during 63 days in weaned lambs of control, selenium nanoparticles and sodium selenite groups

**Fig. 2:**
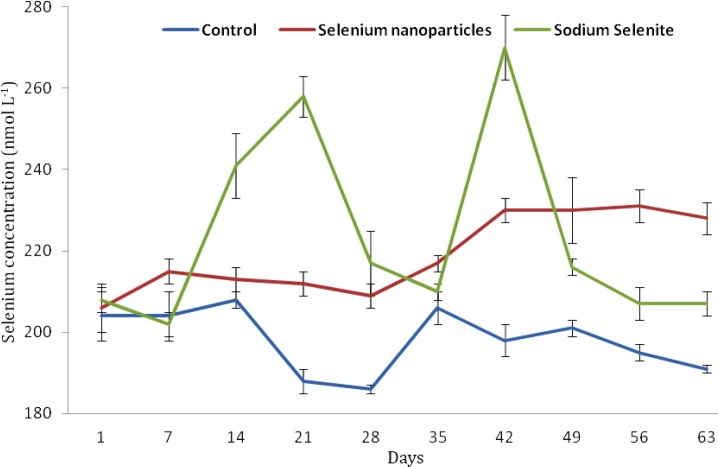
Mean comparison of blood selenium during 63 days in weaned lambs of control, selenium nanoparticles and sodium selenite groups

The overall mean GPX activity, serum Cu and Fe in lambs receiving NaSe and selenium in NanoSe were higher and Zn in lambs receiving NanoSe was lower than other groups (Table 1). Variations in the level of GPX activity (Fig. 1) and selenium (Fig. 2) among groups were significant (*p* < 0.01) but trace mineral levels were not significant among groups (*p* > 0.05). 

Mean comparison of the parameters among 10 times bleeding between groups was significant for GPX activity and selenium (*p* < 0.01) in all groups. Copper was significantly higher and Zn lower in selenium compounds, and not significant for weight gain in all groups (*p* > 0.05), (Table 2). Mean comparison of the parameters within groups was significant in GPX activity, selenium and Cu (*p* < 0.01) in all groups and Fe and weight gain just in lambs receiving NanoSe (*p *< 0.01). 

**Table 1:** Mean for blood minerals, enzyme and weight gain in lambs (n = 40). Data are presented as mean ± standard error (range).

**Parameters**	**Control**	**Selenium nano-particles**	**Sodium selenite**
**Weight (kg)**	24.00 ± 0.45 (18-29)	23.00 ± 0.47 (17-28)	23.60 ± 0.74 (15-32.5)
**Copper (μg dL** ^-1^ **)**	121.00 ± 4.80 (70-193)	126.00 ± 5.30 (55-197)	129.00 ± 4.50 (72-171)
**Zinc (μg dL** ^-1^ **)**	129.00 ± 3.50 (69-221)	120.00 ± 5.20 (61-239)	128.00 ± 3.60 (53-224)
**Iron (μg dL** ^-1^ **)**	96.00 ± 4.00 (52-224)	92.00 ± 4.30(56-197)	101.00 ± 4.20 (61-168)
**GPX (U g** ^-1 ^ **Hb)**	64.00 ± 1.90 (42-86)^a^	92.00 ± 2.50 (63-128)^b^	98.00 ± 7.20 (37-228)^b^
**Selenium (nmol L** ^-1^ **)**	198.00 ± 1.50 (182-220)	219.00 ± 1.90 (195-246)	224.00 ± 5.50 (178-322)

ab Different letters in each row indicate significant differences at *p* < 0.05.

The overall mean weight in control lambs was higher than the selenium compounds but statistically not different with other groups (*p* > 0.05). Mean comparison of the weight gain between and within groups was not different (*p* > 0.05), (Table 2). During the study, lambs gained weight 6.98, 7.60 and 7.33 kg in control, nanoselenium and sodium selenite groups, respectively, which was not different among groups (*p* > 0.05). The percentages of weight gain in three groups was 34.20, 28.90 and 36.30%, respectively which was the same as mentioned in actual weight gain (*p* > 0.05).

Pearson correlations showed no relationships between weight and blood minerals in control lambs and NaSe group but correlations were found between Cu : Zn (r= 0.63, *p* < 0.01), Zn : Fe (r = 0.56, *p *< 0.01) and GPX : Se (r = 0.95, *p *< 0.01) in these groups. Relationships were observed between weight : Fe (r= – 0.46, *p *< 0.01), weight : GPX (r= 0.98, *p* < 0.01) weight : selenium (r= 0.60, *p* < 0.01), GPX : selenium (r= 0.98, *p* < 0.01) and Zn : Fe (r= 0.38, *p* < 0.05) in lambs receiving NanoSe. 

**Table 2:** Mean comparison of weight and blood parameters in lambs (n = 40).

**Parameters**	**Control**			**NanoSe**			**NaSe**		
	**SS**	**MS**	**F-values**	**SS**	**MS**	**F-values**	**SS**	**MS**	**F-values**
**Weight (kg)**	211	24	6.10*	261	29	11.20*	242	27	1.32
**Copper (μg dL** ^-1^ **)**	283123	3146	13.60*	29303	3256	6.77*	25026	2781	13.60*
**Zinc(μg dL** ^-1^ **)**	29327	3259	7.34*	13711	1523	1.60	13092	1455	6.42*
**Iron(μg dL** ^-1^ **)**	9605	1067	2.11†	15471	1719	4.01*	9232	1025	1.76
**GPX (U g** ^-1 ^ **Hb)**	3702	411	7.50*	6054	673	5.90*	75194	8355	50.10*
**Selenium (nmol L** ^-1^ **)**	2125	236	4.66*	3475	386	5.02*	43230	4803	45.80*

SS: Sum of squares, MS: Mean squares.

* indicates significant differences at *p* < 0.01 and

† indicates significant differences at *p* < 0.05.

## Discussion

In this study, the GPX activity in lambs with selenium compounds was greater than the control lambs. The most prominent effect of selenium is to increase the GPX activity and its preventive effects on oxidative stress in animals.^16^ Researchers have proved the antioxidant capacity of selenium compounds in cows,^5^ goats^9^ and sheep^13^ but not in mares.^17^ GPX are responsible for elimination of any active oxygen types in body, and thus play a vital role in the hydrogen peroxide function. Following administration of selenium compounds the GPX activity increases sharply and reaches maximum level within 3 months.^18 ^Decrease in GPX activity causes an increase in hydrogen peroxide level and finally leads to inflammation and tissue damage reflection. Antioxidants are classified as enzymatic and non-enzymatic antioxidants and GPX is the main principle enzymatic antioxidant included selenium mineral.^9^

The mean GPX activity in NaSe group was greater than NanoSe, that is consistent with Shi *et al*.’s study.^8 ^The GPX activity in lambs receiving NaSe showed fluctuation and irregular increase up to the end of the study but in NanoSe groups it was more constant and regular than NaSe administration. The same result has also been reported by others.^9^ Shi *et al*. reported that NanoSe compounds in diet were more efficient than other selenium compounds in terms of antioxidant and GPX activity.^9^ These authors mentioned that GPX activity is possible to increase in blood and simultaneously other tissues such as liver, testicles and semen, too.^9^

An increase in blood selenium following the administration of selenium compounds was also reported by Karren *et al.* and Humann-Ziehanka *et al*.^17^^,^^18^ Blood selenium increased rapidly and continuously after the oral administration of selenium up to the end of study. It was consistent with the result of Shi, *et al*. who mentioned an increase in serum, blood and tissue selenium.^9^ Selenium in the form of selenoenzyme is the major intracellular antioxidant that increases permanence and performance of the body’s cells against any hydrogen peroxides.^12^ Selenoproteins^19^ mainly in combination with vitamin E injection^20^ fortify and amplify the immune and nervous systems function.^21^^,^^22^ Shi *et al.* concluded that NanoSe compounds increase selenium and GPX level more effectively than NaSe and has appropriate indications in sheep.^8^^,^^9^ Meanwhile, the toxicity effect of NanoSe is less than NaSe^10^ and appears as subacute toxicity,^15^ whereas it is acute in NaSe administration.^23^

Mean serum Cu and Fe concentration was higher in NaSe group and Zn was lower in lambs receiving NanoSe, which means that NaSe increases Cu and Fe partially but not significantly in serum. NanoSe decreases serum Zn in lambs, which was in agreement with the results reported by Kojouri and Shirazi.^24^ Moeini *et al*. demonstrated that an increase in selenium in diet decreased Zn serum,^25^ however, Agnieszka and Grażyna described a reduction in all three elements after feeding selenium compounds.^26^ It is necessary that the negative and competitive effect of NanoSe on trace minerals should be reviewed.

The daily mean weight gain in control, NanoSe and NaSe groups was 110.80, 120.60 and 116.30 g, respectively, which indicates partially and not significantly (*p* > 0.05) higher amount of weight gain in selenium supplemented lambs. Studies on the effects of selenium on growth and weight gain of ruminants was widespread but the results did not have a significant effect on growth rate, the same as the results presented in this study.^11^ The result of this study was in agreement with Shi *et al*. reports which mentioned that NanoSe had no effect on growth but improved the average of weight gain,^8^^,^^9^ whereas other researchers have noted a significant weight gain and growth rate in NaSe administration.^11^ The percentage of weight gain in NanoSe lambs was insignificantly (*p* > 0.05) higher than other groups therefore the administration of NanoSe would be the suitable substitution for organic and inorganic selenium compounds in lambs.

The presence of correlations (r = 0.50) among blood minerals in lambs receiving selenium compounds has not been reported yet. The regression equation for the estimation of weight gain in lambs via evaluation of blood GPX and Fe is reported for the first time and needs to be confirmed by scientists. The relationship between weight gain and blood minerals in lambs receiving NanoSe was more reliable and stronger than NaSe, indicating the effectiveness of NanoSe in comparison with organic and inorganic selenium compounds in lambs. The main point in the result of this study was firstly, the strong and close relationship between selenium and GPX activity in lambs receiving selenium compounds, but in the estimation of weight gain in NanoSe group, only GPX and Fe significantly participate in the equation and blood selenium itself was not an important parameter in the weight gain estimation in lambs. Secondly, the correlations among minerals in the selenium compounds group were greater than in control lambs which indicates that selenium and trace minerals may be positively related to each other and should be considered in the administration of mineral supplementation in lambs. 

Finally, selenium compounds increased GPX activity and selenium in which the increases in NanoSe were regular and continuous. Selenium compounds showed no effects on Cu, Zn and Fe but caused partial increase in weight gain. NanoSe revealed the correlations between weight gain, GPX, Fe and selenium and was preferable to NaSe. NanoSe showed regression between weight gain, GPX and Fe that was not observed in NaSe. Thus, the effect of NanoSe in reducing the oxidative stress and increasing weight gain was appropriate and probably supplementing lambs ration with NaSe seems a feasible and effective approach. 
